# Optical Scattering of Liquid Gallium Nanoparticles Coupled to Thin Metal Films

**DOI:** 10.3390/nano10061052

**Published:** 2020-05-30

**Authors:** Fu Deng, Hongfeng Liu, Yuanyuan Peng, Mingcheng Panmai, Sheng Lan

**Affiliations:** Guangdong Provincial Key Laboratory of Nanophotonic Functional Materials and Devices, School of Information and Optoelectronic Science and Engineering, South China Normal University, Guangzhou 510006, China; dengfu@m.scnu.edu.cn (F.D.); scnugd@163.com (H.L.); pengyuanyuan@m.scnu.edu.cn (Y.P.); m.c.panmai@m.scnu.edu.cn (M.P.)

**Keywords:** liquid gallium nanoparticle, scattering, surface plasmon polaritons, radiation pattern

## Abstract

We investigate experimentally and numerically the scattering properties of liquid gallium nanoparticles coupled to a thin gold or silver film. The gallium nanoparticles are excited either directly by using inclined white light or indirectly by surface plasmon polaritons generated on the surface of the gold/silver film. In the former case, the scattering spectrum is always dominated by a scattering peak at ∼540 nm with a long-wavelength shoulder which is redshifted with increasing diameter of the gallium nanoparticle. Under the excitation of the surface plasmon polaritons, optical resonances with much narrower linewidths, which are dependent on the incidence angle of the white light, appear in the scattering spectra. In this case, the scattering spectrum depends weakly on the diameter of the gallium nanoparticle but the radiation pattern exhibits a strong dependence. In addition, a significant enhancement of electric field is expected in the gap region between the gallium nanoparticles and the gold film based on numerical simulation. As compared with the gallium nanoparticle coupled to the gold film which exhibit mainly yellow and orange colors, vivid scattering light spanning the visible light spectrum can be achieved in the gallium nanoparticles coupled to the silver film by simply varying the incidence angle. Gallium nanoparticles coupled to thin metal films may find potential applications in light–matter interaction and color display.

## 1. Introduction

Liquid metals are characterized by their polymorphism, large surface tension, and high electrical conductivity [1,2]. The mechanical and electronic propertities of macroscopic liquid metals have been extensively studied, such as liquid metal enabled pump [3], all-soft matter circuits [4], liquid metal marbles and actuators [5,6], electric-field-induced transformations of liquid metals [7,8], liquid metal mollusks and motors [9,10,11], and self-fueled oscillator machines [12]. Gallium (Ga), a liquid metal at room temperature [13], has attracted tremendous attention because of its peculiar physical and chemical properties [14,15]. The size dependent property of the complex polymorphism of Ga, has also been reported [16]. Ga nanoparticles (NPs) exhibit extreme supercooling and also superheating behavior [13,17,18,19]. Furthermore, the dramatic change in the physical properties of Ga induced by phase transition has been exploited to realize novel nanoscale devices, phase memories [20,21] and active plasmonic platforms [22,23].

During the past decade, the use of metallic nanoparticles (NPs) has been explored in applications of light localization and near field enhancement such as metal-enhanced fluorescence [24] and surface enhanced Raman spectroscopy [25]. Very recently, Ga NPs has been considered as a promising plasmonic material owing to their surface plasmon resonances appearing in the violet spectral range [26]. Analyses of plasmonic NPs often consider isolated metallic spheroids in a homogeneous environment. However, only hemispherical Ga NPs can be obtained when molecular beam epitax (MBE) is employed for the fabrication of Ga NPs [27]. To date, most investigations of the optical properties of Ga NPs were performed on such hemispherical Ga NPs grown by MBE, such as coexistence of liquid and solid phases in substrate-supported Ga NPs [28,29], surface-enhanced Raman spectroscopy, fluorescence and photodegradation [30,31] and deep-subwavelength spectroscopic imaging [32]. Ga NPs can be obtained by using other fabrication methods, such as chemical synthesis [15], electron beam irradiation [33] and light-assisted deposition [20,29]. Unfortunately, a detailed investigation of the optical properties of Ga NPs with spherical shape is still lacking.

To date, the absorption properties of hemispherical Ga NPs have been investigated [27] and the plasmonic behaviors of various hemispherical metallic NPs in near-field and far-field regime have been examined [34]. It has been shown that the resonant interparticle coupling effect in the ultraviolet can be studied by using Mueller matrix ellipsometry [35]. However, there were only a few reports on the optical properties of Ga NPs. Besides, many synthetic Ga NPs were clustered, making it difficult for characterizing the optical properties of an isolated Ga NP [30]. Therefore, characterization of the plasmonic properties of single Ga NPs, especially single Ga NP with spherical shape, has become increasingly important from the viewpoints of both fundamental research and practical application.

Recently, the plasmonic properties of a metallic NP placed on a thin metal film [36,37,38,39,40,41,42,43], has attracted great interest. The plasmonic properties of a gold (Au) NP placed on a thin Au film was numerically studied [36]. Thereafter, the optical properties of an Au NP on an Au film excited by the surface plasmon polaritons (SPPs) generated on the surface of the Au film was experimentally investigated [37]. Moreover, the hybrid gap modes of an Au NP on an Au film were investigated numerically and experimentally [38,39]. However, most of the researches on the plasmonic properties of Ga NPs focus on hemispherical Ga NPs supported by a dielectric substrate [27,30,32,34,35]. The scattering properties of Ga NPs placed on a thin metal film excited by SPPs remain unexplored.

In our recent work, the white light emission from liquid Ga NPs mediated by the Fano resonances in the backward scattering spectra of the Ga NPs placed on an Ag film, which originate from the interference between the mirror-image-induced magnetic dipole mode and the gap plasmon mode, was studied [44]. However, the interaction of the plasmon modes supported by Ga NPs and the propagating SPPs on the surface of a metal film remains unexplored. In this work, we investigated both experimentally and numerically the scattering properties of liquid Ga NPs with different diameters coupled to a thin metal film. These Ga NPs were excited by either inclined white light or the SPPs generated on the surface of the metal film. Forward scattering signals were collected and the radiation patterns were recorded. For Ga NPs located on an Au/silicon dioxide (SiO${}_{2}$) substrate and excited by white light, it was found that the plasmon resonance at short wavelength remained nearly unchanged while that at the long wavelength changed dramatically with increasing diameter. For Ga NPs excited by the SPPs, a single scattering peak with much narrower linewidth, which depends strongly on the angle of the incident light, was observed. The scattering spectrum became not sensitive to the diameter of the Ga NP. Strong enhancement of the electric field is expected in the gap of the Ga NP placed on Au film based on numerical simulation, which may be useful for studying strong light-matter interaction [45,46,47,48]. To gain a deep insight into the plasmonic modes of the Ga NPs coupled to the thin metal film, the scattering spectra and the radiation patterns ware analyzed by using a polarizer with different polarization angles [49,50,51]. Moreover, it was demonstrated that Ga NPs coupled to an silver (Ag) film and excited by the SPPs exhibit a potential application in multicolor display.

## 2. Methods

**Figure 1:**
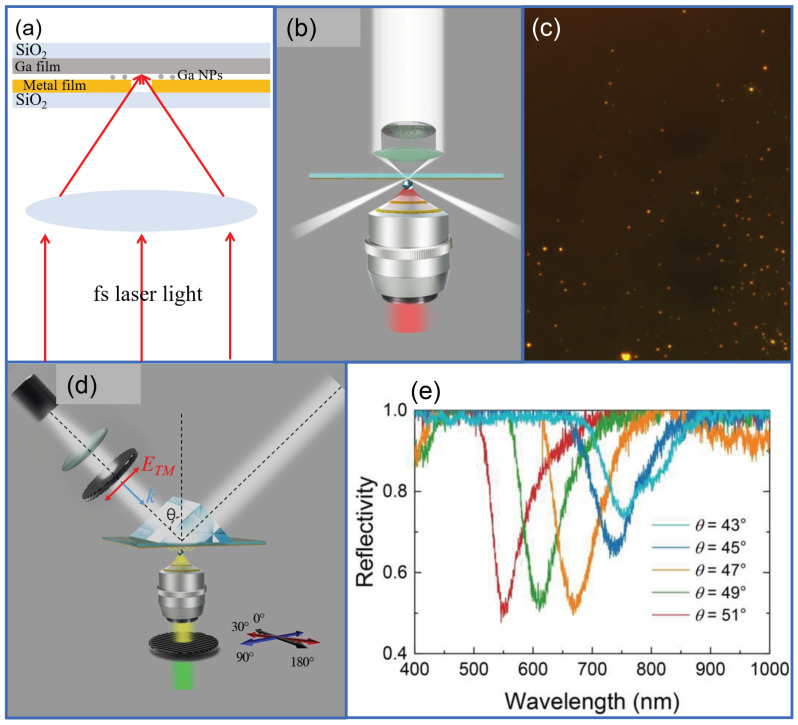
(**a**) Schematic showing the fabrication of Ga nanoparticles (NPs) on a metal/SiO${}_{2}$ substrate by using fs laser ablation of a Ga film. (**b**) Schematic illustrating the measurement of the forward scattering spectrum of a Ga NP coupled to a metal film in a conventional dark-field microscopy. (**c**) Optical image of Ga NPs coupled to an Ag film. (**d**) Schematic illustrating the excitation of a Ga NP coupled to a thin metal by using the surface plasmon polaritons (SPPs) generated on the metal surface via the Kretschmann-Raether (K-R) configuration, and the detection of scattering signal with a polarization analyzer. (**e**) Spectra of the reflected light from the Au film with increasing incidence angle measured in the K-R configuration.

Preparation and characterization of spherical liquid Ga NPs. Ga NPs with different diameters ranging from 150 to 650 nm were fabricated by using femtosecond (fs) laser ablation. A focused fs laser light (Legend, Coherent) with a pulse duration of 100 fs and a repetition rate of 1 kHz was employed to ablate a Ga film in air and the Ga NPs ejected from the Ga film were collected by using a metal/SiO${}_{2}$ substrate, as illustrated in Figure 1a [52]. Such spherical Ga NPs exhibited a core-shell structure with a liquid Ga core and a gallium trioxid (Ga${}_{2}$O${}_{3}$) shell [32]. As shown in Figure 1a, a small hole with a diameter of ∼1.0 mm was initially made on the metal film so that the focused fs laser light can pass through the metal film and arrive at the Ga film. In this way, Ga NPs with different diameters can be obtained on the metal film. The scattering properties of Ga NPs were characterized by using a conventional dark-field microscope (Axio Observer A1, Zeiss, Stutt-gart, Germany) equipped with a spectrometer (SR-500i-B1, Andor, Oxford, UK) and a colour charge coupled device (CCD) (DS-Ri2, Nikonn, Tokyo, Japan) for spectra analysis. The morphologies of the Ga NPs were examined by using scanning electron microscopy (SEM).

Experimental setup. In Figure 1, we show schematically the experimental setups used for measuring the scattering spectra of Ga NPs coupled to thin metal films in air, which are illuminated by white light (Figure 1b) and SPPs generated via the Kretschmann-Raether (K-R) configuration on the surface of the metal film (Figure 1d). A conventional dark-field microscope was employed to measure the forward scattering signal of Ga NPs coupled to the metal film. The optical image of the sample under white light illumination is shown in Figure 1c. When Ga NPs were excited by the SPPs, a polarization analyzer was inserted in the collection channel to filter the scattering light, as shown in Figure 1d. The spectra of the reflected light from the Au film with increasing incidence angle measured via the K-R configuration is shown in Figure 1e. Since the Ga NPs fabricated by using fs laser ablation were generally well separated on the metal/SiO${}_{2}$ substrate, we could perform scattering measurements for single Ga NPs. We usually performed scattering measurements for a large number of Ga nanoparticles located in a specified area which can be easily identified in the scanning electron microscope observations (e.g., with some natural or artificial markers on the sample). After that, the Ga NPs, whose scattering spectra had been obtained, were examined by using SEM. Only Ga NPs with spherical shapes and desirable diameters were used. Therefore, all the scattering spectra presented in the manuscript were obtained from single Ga NPs.

**Figure 2:**
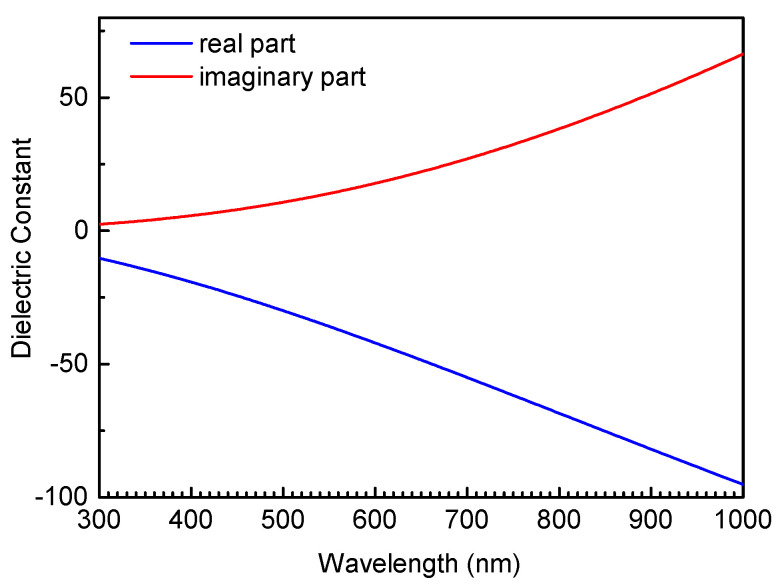
Wavelength dependence of the real and imaginary parts of the complex dielectric constant for liquid Ga used in the calculation of the scattering spectra of Ga NPs.

Numerical Simulations. The scattering properties of Ga NPs were simulated by using the finite-difference time-domain (FDTD) technique. Non-uniform grids with the smallest size of 0.5 nm was used to divide the simulation region which was enclosed by a perfectly matched layer capable of absorbing all outgoing waves in the numerical simulations. The dielectric constants of Ga were taken from the literature [32], as shown in Figure 2. The thickness and refractive index of the Ga${}_{2}$O${}_{3}$ shell were chosen to be 1 nm and 2.2, respectively [53]. The dielectric function of Au was taken from the experimental data [54].

## 3. Results and Discussion

### 3.1. Dark-Field Scattering Properties of Liquid Ga NPs Coupled to an Au Film

**Figure 3:**
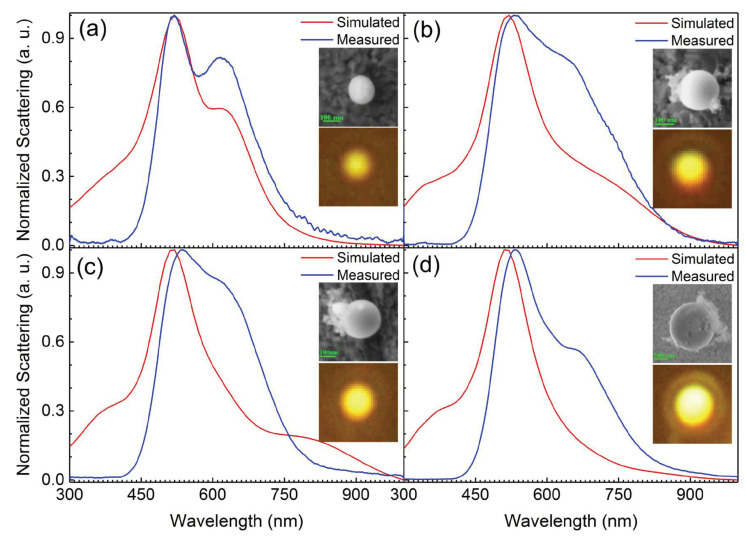
Measured scattering spectra for Ga NPs with different diameters. (**a**) *d* = 165 ± 2 nm, (**b**) *d* = 220 ± 2 nm, (**c**) *d* = 264 ± 2 nm and (**d**) *d* = 657 ± 2 nm. In each case, the SEM images of the Ga NPs and the corresponding radiation patterns are shown as insets. The size of the optical images is 4 × 4 μm${}^{2}$. The simulated scattering spectra are also provided for comparison. All the scattering spectra were normalized in order to compare the spectral shape.

We studied the scattering properties of Ga NPs located on the Au/SiO${}_{2}$ substrate and illuminated by white light firstly, as shown in Figure 1b. The thickness of the Au film was 50 nm. The scattering spectra obtained from a dark-field microscope are shown in Figure 3. In each case, the SEM and CCD images of the corresponding Ga NP are presented in the insets. It was also found that there existed some contamination around or on the surface of some Ga NPs (see SEM images), which influenced the perfectly circular shapes of Ga NPs. However, it hardly influenced the scattering properties of Ga NPs coupled to the Au film, as confirmed by the scattering spectra and radiation patterns. It was found that the scattering spectrum depended strongly on the diameter of the Ga NP. The scattering light appeared to be yellow for Ga NPs with diameters between 150 and 600 nm. It can be seen that the Ga NP with a diameter of *d* = 165 ± 2 nm had two distinct scattering peaks located at ∼520 and ∼615 nm. In comparison, only one scattering peak with a long-wavelength shoulder was observed for Ga NSs with d > 200 nm. With increasing diameter of the Ga NS, the main scattering peak at ∼540 remained nearly unmoved while a redshift of the long-wavelength shoulder was observed. The scattering spectra for the Ga NPs simulated via FDTD technique were also provided. Previously, the scattering properties of Au NPs with different diameters coupled to an Au film were systematically investigated [39]. Although liquid Ga possessed a dielectric function much different from Au, the scattering spectra of the Ga NPs coupled to the Au film appeared quite similar to those observed for Au NPs coupled to an Au film. As shown in Figure 1b, the Ga NP coupled to the Au film was excited by the illumination light transmitted through the thin Au film, which exhibits a transmission peak at ∼530 nm (see Figure S8 in Ref. [39]). Therefore, the scattering peak observed at ∼530 nm arose from the peak transmission of the thin Au film, which remains unchanged with increasing the diameter of the Ga NP. This assignment was confirmed by replacing the Au film with an Ag film whose peak transmission peak appeared at ∼330 nm [39]. In comparison, the scattering peak observed at the longer wavelength was attributed to the radiation of the mirror-image-induced dipole, which originated from the coherent interaction of the electric dipole excited in the Ga NP and its mirror image induced by the Au film [39]. Since the resonant wavelength of the mirror-image dipole is determined by the diameter of the Ga NP (i.e., the separation between the electric dipole and its mirror image), a redshift of the scattering peak was observed with increasing diameter of the Ga NP, as shown in Figure 3.

### 3.2. Au Film-Coupled Liquid Ga NPs Excited by SPPs

**Figure 4:**
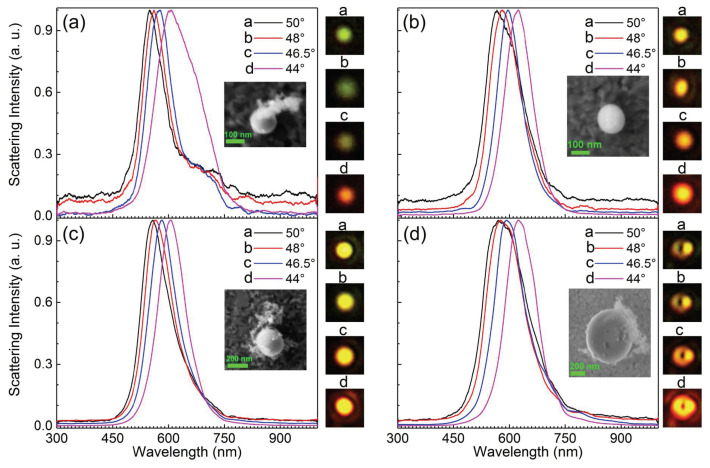
Measured scattering spectra for Ga NPs with different diameters on the Au/SiO${}_{2}$ substrate, which are excited by the SPPs generated at different incidence angles. (**a**) *d* = 146 ± 2 nm, (**b**) *d* = 165 ± 2 nm, (**c**) *d* = 281 ± 2 nm and (**d**) *d* = 657 ± 2 nm. In each case, the SEM images of the Ga NPs and the corresponding radiation patterns are shown as insets. The size of the optical images is 4 × 4 μm${}^{2}$. All the scattering spectra have been normalized in order to compare the spectral shape.

In our previous work, we successfully fabricated liquid Ga NPs with different diameters in water via fs laser ablation [44]. It was found that the scattering spectra of liquid Ga NPs spanned the visible light to the near-infrared spectral range. This unique feature makes it possible to use large Ga NPs in combination with a thin metal film for constructing plasmonic nanocavities with significantly enhanced electric field. Such nanocavities with embedded two-dimensional materials are suitable for studying strong plasmon-exciton coupling because the broad scattering spectra ensure the clear identification of energy splitting while the large sizes enhance the signal-to-noise ratio [48]. In this work, the scattering properties of a Ga NP excited by the propagating SPPs generated on the Au film surface were experimentally studied. We examined the scattering spectra of Ga NPs with *d* = 146 ± 2 nm, 165 ± 2 nm, 281 ± 2 nm and 657 ± 2 nm placed on an Au/SiO${}_{2}$ substrate. In Figure 4, we present the scattering spectra measured for the Ga NPs with different diameters excited by using the SPPs generated at different incidence angles of θ = 44${}^{\circ }$, 46.5${}^{\circ }$, 48${}^{\circ }$, and 50${}^{\circ }$. When the incidence angle was changed from 44${}^{\circ }$ to 50${}^{\circ }$, SPPs with different plasmon wavelengths were generated on the Au film surface. For the Ga NP with *d* = 146 ± 2 nm, the color of the scattering light was changed from red to yellow and finally to green (see Figure 4a) with increasing the incidence angle. For Ga NPs with larger diameters, only the change of the scattering light color from red to yellow was observed (see Figure 4b–d). For the Ga NP with *d* = 657 ± 2 nm, a broadening of the scattering spectrum appeared, especially at small incidence angles. In this case, a doughnut-shaped radiation pattern was observed, possibly due to the high-order plasmon modes.

**Figure 5:**
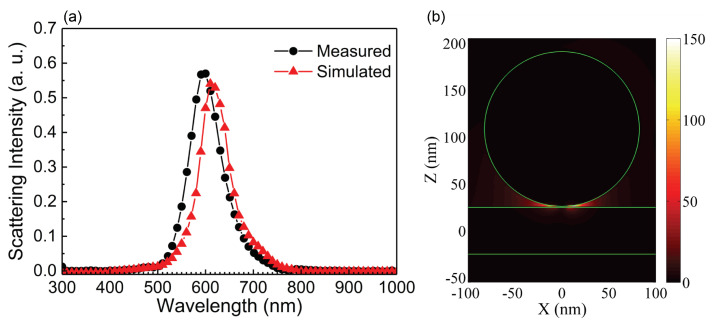
(**a**) Measured and calculated scattering spectra for the Ga NP with *d* = 165 ± 2 nm which was excited by the SPPs generated at an incidence angle of θ = 46.5${}^{\circ }$. (**b**) The calculated XZ plane electric field distribution |E| for the Ga NP at the scattering peak.

To gain a deep insight into the plasmonic properties of the Ga NPs, the scattering spectra of the Ga NPs were also simulated by using FDTD simulation, as shown in Figure 5 where the comparison of the scattering spectra measured and calculated for the Ga NP with *d* = 165 ± 2 nm is provided. It can be seen that a very good agreement between the experimental observation and the simulation result was achieved. Additionally, the corresponding electric field distribution at the scattering peak is shown in Figure 5b. A enhancement as large as ∼150 was observed at the gap region between the Ga NP and the Au film, implying the feasibility of using such a nanocavity with an embedded two-dimensional material for investigating strong plasmon-exciton coupling [45,46,47]. In this case, the two-dimensional materials could be grown on a sapphire substrate and then transferred to the Au film [48]. Ga NPs with different diameters could be fabricated in water and dispersed on the two-dimensional materials by drop-casting [44]. Basically, the electric field enhancement was determined by the contacting point between the Ga NP and the Au film. Although a spherical Ga NP was used to simulate the electric field distribution, it was confirmed that a similar enhancement factor for the electric field could be achieved even though the spherical Ga NP was changed to an ellipsoidal one. Based on numerical simulation, it was found that the core-shell structure of Ga NPs had negligible on their scattering spectra. However, thickness of the shell affected the electric field enhancement achieved in the gap region.

**Figure 6:**
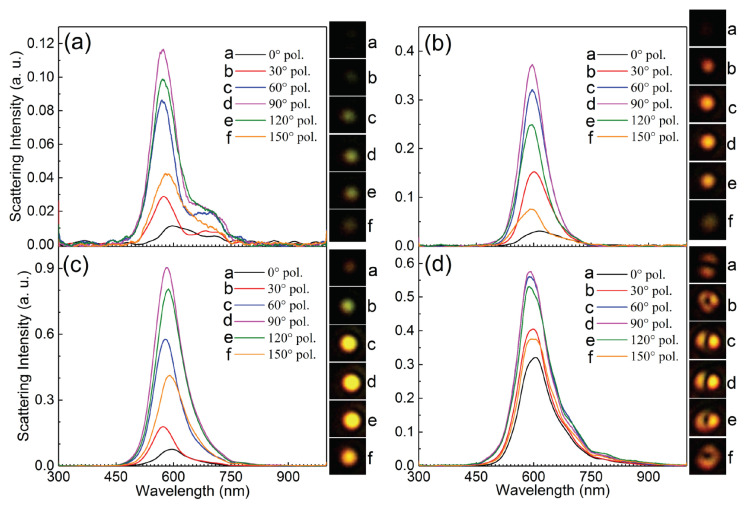
Polarization-resolved scattering spectra measured for Ga NPs with different diameters placed on the Au/SiO${}_{2}$ substrate (**a**) *d* = 146 ± 2 nm, (**b**) *d* = 165 ± 2 nm, (**c**) *d* = 281 ± 2 nm and (**d**) *d* = 657 ± 2 nm. The corresponding radiation patterns are shown in the insets and the size of the optical images is 4 × 4 μm${}^{2}$.

To identify the plasmon modes in Ga NPs excited by SPPs, a polarization analyzer was inserted into the collection channel to filter the scattering light, acquiring polarization-resolved scattering spectra and radiation patterns. In Figure 6, we show the polarization-resolved scattering spectra measured for Ga NPs with different diameters. The corresponding radiation patterns were also recorded, as shown in the insets. For Ga NPs with *d* = 146 ± 2, 165 ± 2 and 281 ± 2 nm, the strongest scattering intensity was found at a polarization angle of 90${}^{\circ }$, whose direction was the same as the propagation direction of the SPPs. The scattering intensity was almost one order of magnitude larger than that in the cross-polarization direction (i.e., 0${}^{\circ }$). It implies that the radiation of the Ga NP was dominated by the dipole moment oriented along 90${}^{\circ }$. For the Ga NP with *d* = 657 ± 2 nm, the difference between the scattering intensity at 90${}^{\circ }$ and that at 0${}^{\circ }$ was reduced dramatically. In addition, a doughnut-shaped radiation pattern was observed for this Ga NP, implying that the radiation was dominated by high-order plasmon mode rather than dipole mode. Therefore, the rotation of the polarization analyzer did not change the scattering intensity so much. Accordingly, one can clearly see the rotation of the polarization analyzer in the radiation pattern shown in the insets.

### 3.3. Ag Film-Coupled Ga NPs for Color Display

**Figure 7:**
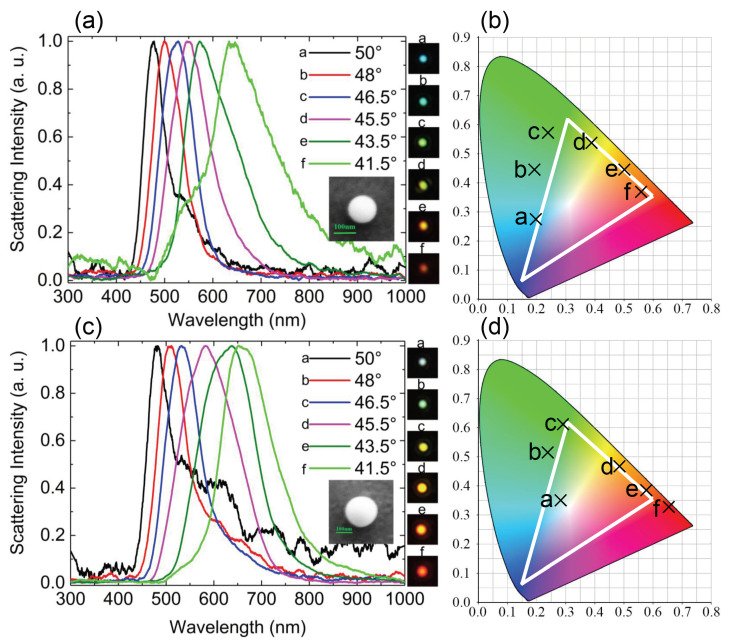
Measured scattering spectra of two Ga NPs with different diameters placed on the SiO${}_{2}$–SnO${}_{2}$/Ag/SiO${}_{2}$ substrate. (**a**) *d* = 124 ± 2 nm and (**c**) *d* = 187 ± 2 nm. (**b**,**d**) are the color indices derived from the scattering spectra shown in (**a**,**c**). The SEM images of the Ga NPs and the corresponding radiation patterns are shown as insets in (**a**,**c**). The size of the optical images is 4 × 4 μm${}^{2}$.

An intriguing phenomenon exhibited by Ga NPs is the possibility for engineering the scattering light through coupling to different thin metal films. As an example, we show here the scattering properties of Ga NPs coupled to an Ag film and also excited by the SPPs. We replaced the Au/SiO${}_{2}$ substrate used above by a SiO${}_{2}$-SnO${}_{2}$/Ag/SiO${}_{2}$ substrate where a 50-nm-thick Ag film was covered with an anti-oxidation dielectric layer (SiO${}_{2}$-stannic dioxide (SnO${}_{2}$)) with a thickness of 10 nm and a refractive index of ∼1.7. Similarly, we can see the redshift of the scattering peak from 475 to 650 nm, which spanned almost the entire visible light spectrum, when the incidence angle was varied, as shown in Figure 7a,c. It is remarkable that the linewidths of the scattering spectra appeared to be much narrower than those observed for Ga NPs located on the Au/SiO${}_{2}$ substrate, implying good chromativity for color display. These features suggest that Ga NPs coupled with an Ag film may be employed to realize nanoscale multicolor display with high spatial resolution, wide color tuning range and good chromativity, as shown in Figure 7b,d where the color indices obtained at different incidence angles are presented. It is found that the color indices in this case were distributed around the RGB triangle, implying good chromaticity of the structural color produced by the Ga NPs coupled to the Ag film.

## 4. Conclusions

In conclusion, we have investigated experimentally and numerically the scattering properties of liquid Ga NPs coupled to thin metal films and excited by white light and SPPs. As compared with the Ga NPs excited by white light which generally exhibit broad scattering spectra and yellow scattering light, the Ga NPs excited by SPPs generate narrow scattering spectra and vivid scattering light, especially for those located on an Ag film rather than an Au film. The scattering spectra of the Ga NPs excited by white light exhibit a strong dependence on the diameter of the Ga NP while their scattering patterns remain unchanged with increasing diameter of the Ga NP. In sharp contrast, the scattering spectra of the Ga NPs excited by SPPs depend strongly on the angle of the incident light and weakly on the diameter of the Ga NP. In addition, an apparent change in the scattering pattern is observed for Ga NPs with large diameters. A large enhancement in the electric field is achieved at the gap region between the Ga NP and the metal film, which could be exploited for realizing strong light-matter interaction. Our findings are helpful for understanding the mode hybridization in Ga NPs coupled to metal films and useful for designing nanoscale devices for light-matter interaction and color display.

## Abbreviations

The following abbreviations are used in this manuscript:

Ga	gallium
Au	gold
Ag	silver
SPPs	surface plasmon polaritons
NP	nanoparticle
MBE	molecular beam epitaxy
K-R	Kretschmann-Raether
SEM	scanning electron microscopy
CCD	charge coupled device
FDTD	finite-difference time-domain
Ga${}_{2}$O${}_{3}$	gallium trioxide
SiO${}_{2}$	silica
SnO${}_{2}$	stannic dioxide
